# Variable Selection from Image Texture Feature for Automatic Classification of Concrete Surface Voids

**DOI:** 10.1155/2021/5538573

**Published:** 2021-03-06

**Authors:** Ziting Zhao, Tong Liu, Xudong Zhao

**Affiliations:** ^1^College of Civil Engineering, Northeast Forestry University, Harbin 150040, China; ^2^College of Information and Computer Engineering, Northeast Forestry University, Harbin 150040, China

## Abstract

Machine learning plays an important role in computational intelligence and has been widely used in many engineering fields. Surface voids or bugholes frequently appearing on concrete surface after the casting process make the corresponding manual inspection time consuming, costly, labor intensive, and inconsistent. In order to make a better inspection of the concrete surface, automatic classification of concrete bugholes is needed. In this paper, a variable selection strategy is proposed for pursuing feature interpretability, together with an automatic ensemble classification designed for getting a better accuracy of the bughole classification. A texture feature deriving from the Gabor filter and gray-level run lengths is extracted in concrete surface images. Interpretable variables, which are also the components of the feature, are selected according to a presented cumulative voting strategy. An ensemble classifier with its base classifier automatically assigned is provided to detect whether a surface void exists in an image or not. Experimental results on 1000 image samples indicate the effectiveness of our method with a comparable prediction accuracy and model explicable.

## 1. Introduction

Machine learning plays an important role in computational intelligence. Many learning classifiers (e.g., support vector machine, fuzzy *k*-nearest neighboring, and neural networks with fuzzy systems) and intelligent algorithms have been used for computer-aided systems including adaptive force control of machining processes [1], fuzzy control of energy equipment [2], ultraprecision cascade control of mechanical device [3], and even object detection (e.g., obstacle classification [4]). Object classification in civil engineering is also a case in point.

Surface voids or bugholes, which are derived from the migration of an entrapped air bubble to the interface between fresh concrete and formwork [5] are considered to be one of the most seriously encountered defects on the concrete surface [6]. Visually, they correspond to scattered small pits and craters on the concrete surface after the process of formwork removal [7]. The existence of surface voids leads to many flaws, which are listed as follows. A certain amount of bugholes on concrete surface may leave an unaesthetic impression [8]. Reinforcements inside may be exposed and corroded due to the surface voids [5]. Excessive voids may reduce the adhesion properties of the fiber-reinforced plastic materials applied to the concrete surface [9]. Premature degradation of reinforced concrete structures may occur due to salt accumulation in surface voids [10].

Traditional methods for classification of surface voids are based on manual inspection of the concrete surface [11–13] or on manual comparison between the concrete surface and a set of standard surface photographs of reference samples [6, 8]. Such methods are not only time consuming and costly but also labor intensive and inconsistent. Hence, it makes the automatic methods for recogniton of surface voids mainstream for assessing the inspection result of the concrete surface, which are less time consuming, nonexpensive, technology intensive, and objective, comparatively.

In order to get a better evaluation of the concrete surface, many image processing technologies and machine learning methods have been utilized. As to classification of concrete surface voids, there are mainly three different kinds of automatic classification approaches. The first kind of the methods refers to image thresholding or filtering for segmentation of concrete surface voids. Liu and Yang [14] used a pixel-level gray threshold segmentation method for classification of surface voids. Zhu and Brilakis [15] created a spatial spot filter convolved with the concrete surface to detect surface voids. da Silva and Štemberk [16] employed Winer's filter to reduce the noise and a morphological filter to enhance the image contrast, which provided optimized segmentation results of concrete surface voids. Although these methods can obtain the areas or the edges of concrete surface voids rapidly, the accuracy of classification results is still constrained by the complexity of the heterogeneous concrete surface background and varying lighting conditions.

Therefore, prevailing research studies convert to the extraction of texture features from an image of concrete surface, considering the insensitivity of texture to illumination. In addition, different classification models (namely, classifiers) for discriminating whether there are bugholes or not on the concrete surface are always presented. Typically, Hoang and Nguyen [17] employed the Gabor filter and gray-level run lengths to generate a 108-dimensional texture feature derived from a concrete surface. Besides, a support vector machine (SVM) with its parameters optimized by employing an adaptive differential evolution with linear population size reduction was utilized to classify images of concrete surfaces into ones with surface voids and the others without surface voids. Anyway, a complex hybrid of different texture features and classifiers needs to be tried to obtain better classification accuracies corresponding to certain images of the concrete surface.

In order to simply raise the accuracy of the bughole classification, a deep learning-based convolution neural network (CNN) [18] needs to be considered. Yao et al. [19] extended the CNN by designing the inception modules to detect bugholes on concrete surfaces. In addition, they [20] presented an instance-level method for classification of concrete surface bugholes based on Mask R-CNN. These research studies demonstrate a great capability of the CNN in identifying concrete surface voids accurately. However, training a CNN often needs a large number of training images and a large computational overhead. Besides, CNN models commonly lack of interpretation to classification results. In order to represent a compromise between the interpretability and high accuracies of the bughole classification, traditional methods that put forward feature selection before classifiers are to be re-examined.

In this paper, we propose a variable selection method for automatic classification of surface voids from concrete images. Variables which are considered as the components of the 108-dimensional texture feature [17] are selected using a presented cumulative voting strategy. After that, ensemble classification is made on the selected variables to distinguish the images of concrete surfaces with bugholes and those without surface voids. The selected variables support better interpretability. As to ensemble classification, it provides high classification accuracies. The remaining parts of this paper are as follows: in Section 2, we indicate the related texture feature and present a variable selection strategy; in Section 3, the used data are described, and corresponding experimental results are shown and explained; in Section 4, discussion is provided; and the conclusion and prospect are given in Section 5.

## 2. Methods

### 2.1. Related Texture Feature

In order to support the surface void classification process, texture feature which is insensitive to illumination is always considered. Following Hoang and Nguyen's research work [17], the Gabor filter and gray-level run-length methods are employed for feature extraction.

Commonly, surface voids are regarded as abnormal regions of a concrete surface with regular texture. A Gabor filter is considered to be an effective approach for texture discrimination [21]. The response of the symmetric Gabor filter is expressed as

(1) $$\begin{array}{c} g\left(x,y\right)=\exp\left\{-\frac{1}{2}\left[\frac{x^{2}}{\sigma_{x}^{2}}+\frac{y^{2}}{\sigma_{y}^{2}}\right]\right\}\cos\left(2\pi u_{0}x\right), \end{array}$$

where *u*_0_ represents the frequency at which Gabor filter responds most strongly along the *x*-axis. *σ*_*x*_ and *σ*_*y*_ denote the spatial scaling coefficient along the *x*- and *y*-axis, respectively. The corresponding frequency transformation is

(2) $$\begin{array}{c} G\left(u,v\right)=A\left(\exp\left\{-\frac{1}{2}\left[\frac{{\left(u-u_{0}\right)}^{2}}{\sigma_{u}^{2}}\right]+\frac{v^{2}}{\sigma_{v}^{2}}\right\}+\exp\left\{-\frac{1}{2}\left[\frac{{\left(u+u_{0}\right)}^{2}}{\sigma_{u}^{2}}\right]+\frac{v^{2}}{\sigma_{v}^{2}}\right\}\right), \end{array}$$

where *σ*_*u*_, *σ*_*v*_, and *A* are expressed as *σ*_*u*_=(1/2)*πσ*_*x*_, *σ*_*v*_=(1/2)*πσ*_*y*_, and *A*=2*πσ*_*x*_*σ*_*y*_, respectively. It has been stated that the tuning parameters of the Gabor filter including the orientation angles and the radial frequencies must be specified in order to better recognize texture [22]. As suggested in the previous work of Hoang and Nguyen [17], the orientation angles including 0°, 45°, 90°, and 135° can be used. As to the radial frequency *u*_0_, it can be set to $1\sqrt{2}$, $2\sqrt{2}$, $4\sqrt{2}$,…, $\left(N_{w}/4\right)\sqrt{2}$, where *N*_*w*_ represents the number of pixels and is a power of 2.

Gray-level run-length refers to a pattern of gray-intensity pixels in a particular direction from a reference point [23]. Given a certain direction in an image (e.g., 0°, 45°, 90°, and 135°), a run-length matrix *p*(*i*, *j*) is composed of the run-length *j* of gray level *i*. Based on the run-length matrix, Hoang and Nguyen [17] employed a variety of statistics to describe texture in the image. In turn, these statistics include short-run emphasis (SRE), long-run emphasis (LRE), gray-level nonuniformity (GLN), run-length nonuniformity (RLN), run percentage (RP), low gray-level run emphasis (LGRE), high gray-level run emphasis (HGRE), short-run low gray-level emphasis (SRLGE), short-run high gray-level emphasis (SRHGE), long-run low gray-level emphasis (LRLGE), and long-run high gray-level emphasis (LRHGE), which are listed as follows:

(3) $$\begin{array}{c} \text{SRE}=\frac{1}{N_{r}}\sum_{i=1}^{M}\sum_{j=1}^{N}\frac{p\left(i,j\right)}{j^{2}},\\ \text{LRE}=\frac{1}{N_{r}}\sum_{i=1}^{M}\sum_{j=1}^{N}p\left(i,j\right)\times j^{2},\\ \text{GLN}=\frac{1}{N_{r}}\sum_{i=1}^{M}{\left(\sum_{j=1}^{N}p\left(i,j\right)\right)}^{2},\\ \text{RLN}=\frac{1}{N_{r}}\sum_{j=1}^{N}{\left(\sum_{i=1}^{M}p\left(i,j\right)\right)}^{2},\\ \text{RP}=\frac{N_{r}}{N_{p}},\\ \text{LGRE}=\frac{1}{N_{r}}\sum_{j=1}^{N}\sum_{i=1}^{M}\frac{p\left(i,j\right)}{i^{2}},\\ \text{HGRE}=\frac{1}{N_{r}}\sum_{j=1}^{N}\sum_{i=1}^{M}p\left(i,j\right)\times i^{2},\\ \text{SRLGE}=\frac{1}{N_{r}}\sum_{j=1}^{N}\sum_{i=1}^{M}\frac{p\left(i,j\right)}{i^{2}\times j^{2}},\\ \text{SRHGE}=\frac{1}{N_{r}}\sum_{j=1}^{N}\sum_{i=1}^{M}\frac{p\left(i,j\right)\times i^{2}}{j^{2}},\\ \text{LRLGE}=\frac{1}{N_{r}}\sum_{j=1}^{N}\sum_{i=1}^{M}\frac{p\left(i,j\right)\times j^{2}}{i^{2}},\\ \text{LRHGE}=\frac{1}{N_{r}}\sum_{j=1}^{N}\sum_{i=1}^{M}p\left(i,j\right)\times i^{2}\times j^{2}, \end{array}$$

where *M* and *N*_*p*_ denote the number of gray levels and pixels, respectively. *N*_*r*_ and *M* represent the total number of runs and the maximum run length, respectively.

As stated in the previous work of Hoang and Nguyen [17], four orientation angles (i.e., 0°, 45°, 90°, and 135°) and four radial frequencies (i.e., $1\sqrt{2}$, $2\sqrt{2}$, $4\sqrt{2}$, and $8\sqrt{2}$) are employed to obtain 16 filtered images. As to each filtered image, four statistics representing the mean, standard deviation, skewness, and entropy of the Gabor filter response are calculated. That is,

(4) $$\begin{array}{c} \text{Mean}=\frac{\sum_{i=0}^{H-1}\sum_{j=0}^{W-1}\text{GFR}\left(i,j\right)}{W\times H},\\ \text{STD}=\frac{\sum_{i=0}^{H-1}{\sum_{j=0}^{W-1}\left[\text{GFR}\left(i,j\right)-\text{Mean}\right]}^{2}}{W\times H},\\ \text{Skewness}=\frac{\left[1/\left(W\times H-1\right)\right]\sum_{i=0}^{H-1}\sum_{j=0}^{W-1}{\left[\text{GFR}\left(i,j\right)-\text{Mean}\right]}^{3}}{{\left\{\left[1/\left(W\times H-1\right)\right]\sum_{i=0}^{H-1}\sum_{j=0}^{W-1}{\left[\text{GFR}\left(i,j\right)-\text{Mean}\right]}^{2}\right\}}^{3/2}},\\ \text{Entropy}=-\sum_{i=0}^{255}\text{FOH}\times \;\log_{2}\text{FOH}, \end{array}$$

where GFR(*i*, *j*) represents the Gabor filter response at pixel (*i*, *j*). *H* and *W* are the height and width of the image, respectively.FOH is the first-order histogram of the Gabor filter response. As a result, 64 components of the texture feature are derived from Gabor filtering. As to gray-level run-length, four orientations (i.e., 0°, 45°, 90°, and 135°) of the 11 statistics shown in equation (3) form 44 components of the texture feature. Together, they constitute the 108-dimensional texture feature for classification of surface voids. The extracted feature is entirely used to classify images of concrete surfaces into ones with surface voids and the others without surface voids. In fact, it may be only parts of the feature are considered to be effective for further classification.

### 2.2. The Variable Selection Strategy

**Figure 1:**
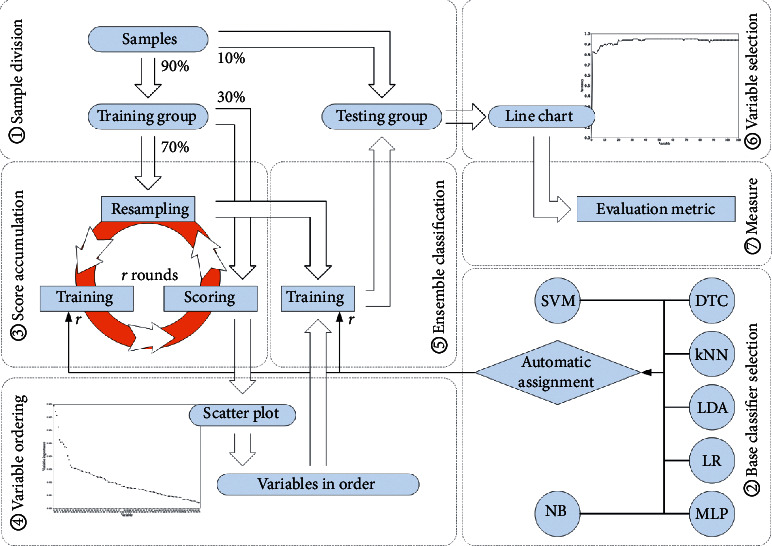
A framework of the variable selection strategy.

Here, we introduce a cumulative voting strategy for selection of variables [24] corresponding to the 108 components of the extracted feature. The proposed variable selection strategy is divided into seven steps each of which is framed and labeled in a dashed box, as is shown in Figure 1.

At the first step, samples representing concrete surface images with or without bugholes are randomly divided. That is, 90% of the samples are randomly chosen to constitute a training group, while the left samples compose a testing group.

At the second step, a base classifier is automatically assigned from seven base classifiers including support vector machine (SVM) decision tree classifier (DTC), k-nearest neighbor (kNN), linear discriminant analysis (LDA), logistic regression (LR), multilayer perceptron (MLP), and naive Bayesian (NB). In each round *k*, 70% of training samples are randomly selected for training each base classifier in 108-dimensional feature space. The remaining 30% of training samples are used to calculate the classification error rate Err_*k*_, which is expressed as

(5) $$\begin{array}{c} \text{Err}=\frac{\text{FN}+\text{FP}}{\text{TP}+\text{FN}+\text{TN}+\text{FP}}, \end{array}$$

where TP, TN, FP, and FN denote the number of true positive, true negative, false positive, and false negative, respectively. Positive samples refer to images with surface voids, while negative samples correspond to images without surface voids. The base classifier which keeps the lowest classification error rate is automatically assigned in this round.

Score accumulation is made at the third step. Permutations are performed after the automatic assignment of a base classifier in round *k*. As to each variable *c*, only one-time permutation of the component values from the 30% of left samples is made. The corresponding classification error rate is expressed as Err_*k*_^0^(*c*). Accordingly, a score representing the importance of the variable *c* is denoted as score_*k*_(*c*)=Err_*k*_^0^(*c*) − Err_*k*_. After *r* rounds of resampling, training, and scoring, the accumulated score of the variable *c* is expressed as ∑_*k*=1_^*r*^(score_*k*_(*c*)/*r*).

Variables are reordered at the fourth step. A 2D scatter plot is made with its *x*- and *y*-axis corresponding to the variable indices and the accumulated scores, respectively. If the accumulated scores of the variables are all relatively low, all the variables but not the significant ones selected using previously proposed clustering approach [25] are to be used at the following steps.

Ensemble classification is performed at the fifth step. *r* rounds of resampling and training are made to establish ensemble classifiers in each dimension according to variables incrementally added in a descending order according to the accumulated scores. At each round of resampling, the base classifier with the lowest classification error rate is trained. This procedure is repeated from one to all the 108 dimensions, with a variable keeping a lower accumulated score added at each time.

Variable selection is made at the sixth step. In each dimension, the established ensemble classifier is applied to the testing samples. The accuracy (Acc) is calculated and expressed as

(6) $$\begin{array}{c} \text{Acc}=\frac{\text{TN}+\text{TP}}{\text{TP}+\text{FN}+\text{TN}+\text{FP}}. \end{array}$$

Accordingly, a line chart is obtained with its *x*- and *y*-axis corresponding to the variable indices in the descending order and the corresponding Accs in different dimensions. Therefore, a dimension threshold can be made when Accs keep almost the same with dimension incrementally increasing. That makes variables which are helpful to recognize surface voids from concrete surface images selected out from the 108-dimensional feature.

At the seventh step, evaluation metrics are made to estimate the effectiveness of the selected variables. In addition to Acc, we also choose three widely used quantitative measurements. That is,

(7) $$\begin{array}{c} \text{Precision}=\frac{\text{TP}}{\text{TP}+\text{FP}},\\ \text{Recall}=\frac{\text{TP}}{\text{TP}+\text{FN}},\\ F1-\text{measure}=\frac{2\times \text{Precision}\times \text{Recall}}{\text{Precision}+\text{Recall}}. \end{array}$$

## 3. Results

### 3.1. Image Samples of Concrete Surface

**Figure 2:**
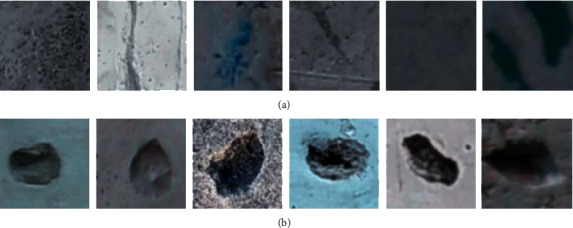
The collected image samples. (a) Nonsurface void and (b) surface void.

We use a set of 1000 image samples capturing the texture of concrete structures provided by Hoang and Nguyen [17] for comparison, which is downloaded from the repository of github (https://github.com/NhatDucHoang/L-SHADE-SVM-SVD). In total, these 1000 images include 500 positives having surface voids and 500 negatives without surface voids, each of which keeps an image size of 20 × 20 pixels (see Figure 2). A 108-dimensional feature is extracted from each image. *Z*-score normalization is made on each feature derived from an image. Then, the 1000 samples are randomly divided. 90% of the samples are randomly chosen as a training group for model construction, and the remaining 10% are regarded as a testing group for evaluating the model performance. Sample division has been made 20 times, in order to diminish the effect on evaluating the predictive capability due to random sample selection. Our method is developed in Python3.8 with skleanV0.24 and numpyV1.19. The corresponding program runs in a PC (Core i7-6700, 8 G RAM, 256 GB solid-state drive). A previously proposed variable selection tool [26] can be alternatively used.

### 3.2. Results of Base Classifier Selection

**Figure 3:**
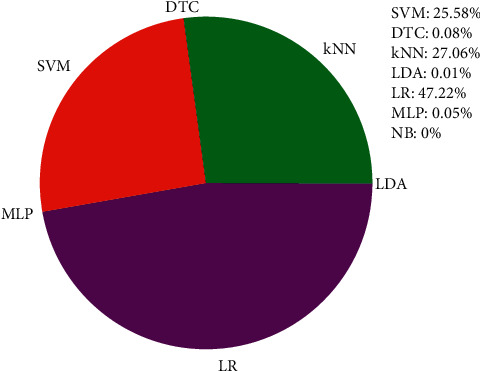
A pan chart showing the proportion of the selected base classifiers after 10,000 rounds of resampling and training at the base classifier selection step during one-time sample division.

In each time of sample division, we make 10000 rounds of resampling and training. In each round, a base classifier keeping the lowest classification error rate is automatically assigned. Default parameters of the seven base classifiers are used. After 10000 rounds, the proportion of selected base classifiers is illustrated in Figure 3. It can be seen that LR is automatically selected for 4722 times. kNN and SVM come in second and third places with 27.06% and 25.58% of the 10000 round automatic selection of base classifiers. DTC, MLP, LDA, and NB are automatically selected in a descending order with their selected times staying in single digits. The experimental results indicate that LR, kNN, and SVM are the appropriate classifiers for discrimination between positive samples and negative ones in 108-dimensional feature space.

### 3.3. Results of Score Accumulation and Variable Ordering

**Figure 4:**
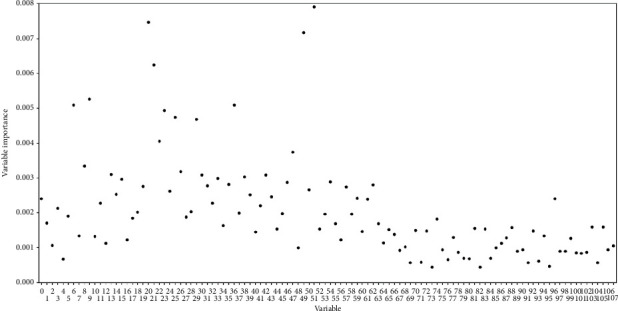
A scatter plot which illustrates accumulated scores (i.e., variable importance) and the corresponding variables during one-time sample division.

As to the selected base classifier in each round, one-time permutation on each variable of the 108-dimensional feature is made, and a score representing the importance of the variable is calculated. In each round of resampling, training, and scoring, the score of each variable is accumulated. The accumulation is ended after 10000 rounds. Then, the 108 variables are reordered according to their accumulated scores in a descending order. As shown in Figure 4, the accumulated scores are all relatively low with the highest score being 0.008. In that case, all the variables have to be considered.

### 3.4. Results of Ensemble Classification and Variable Selection

**Figure 5:**
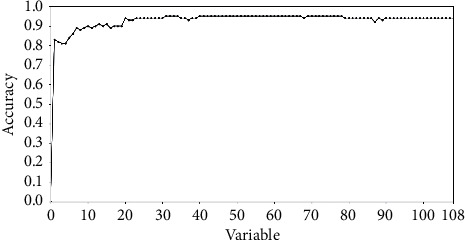
A line chart showing accuracies in different dimensions and corresponding variables reordered according to their accumulated scores in a descending order during one-time sample division.

We make an incremental strategy on all the variables. That is, variables are added one by one to establish features from one dimension to 108 dimensions following their importance ranging in a descending order. In each dimension, an ensemble classifier is built after 1000 rounds of resampling and training. Then, the established ensemble classifier is applied to the 10% of independent samples for testing. Thus, the accuracies in each dimension are obtained as shown in Figure 5. That is, a line chart is obtained with its *x*- and *y*-axis representing variables in the descending order, and the corresponding accuracies are calculated using equation (6) in different dimensions. It can be seen in Figure 5 that it is the first 20 variables with descending accumulated scores that form a 20-dimensional feature comparable to the feature with 108 components for effective classification of surface voids in concrete images.

### 3.5. Classification Results of the Selected Variables

**Figure 6:**
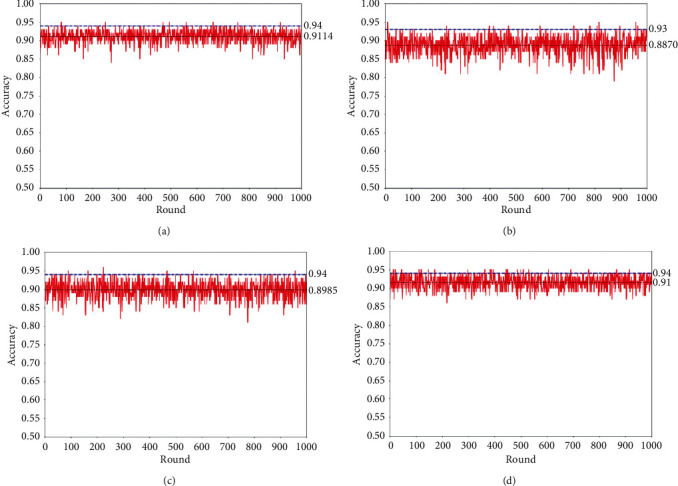
Comparative experimental results on the selected 20 variables during one-time sample division. Red solid lines show the accuracies of a base classifier on 30% of testing samples in each round of resampling. Black dotted lines illustrate the corresponding mean accuracy values of the 1000 rounds of resampling. Blue dotted dash lines show the accuracies on 10% of independent testing samples. (a) The experimental results on the selected 20 variables. (b) The experimental results on 1000 rounds of random variable selection in 20 dimensions from the 21th variable to the 108th one. (c) The experimental results on 1000 rounds of random variable selection in 20 dimensions from all the 108 components. (d) The experimental results on all the 108 variables.

In order to illustrate the effectiveness of the selected 20 variables, experiments are made as shown in Figure 6. Figures 6(a)–6(d) illustrate the classification accuracies of the selected 20 variables, any 20 variables extracted from the 21th to the 108th components, any 20 variables extracted from the 108 components, and all the 108 variables, respectively. It can be seen that the selected 20 variables keep higher mean accuracies on 30% of the left samples in each round of resampling, i.e., 0.9114, and a comparable accuracy on 10% of the independent samples, i.e., 0.94.

**Figure 7:**
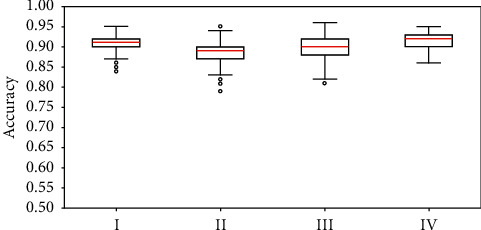
Blot plots. I II, III, and IV refer to the experimental results of the selected 20 variables, 1000 rounds of random variable selection in 20 dimensions from the 21th variable to the 108th one, 1000 rounds of random variable selection in 20 dimensions from all the 108 variables, and all the 108 variables. The red lines refer to the median values.

We also make the corresponding blot plots as shown in Figure 7. Here, I, II, III, and IV correspond to the experimental results shown in Figures 6(a)–6(d), respectively. It can be seen that the selected 20 variables can obtain a more stable accuracy at a high level.

### 3.6. Classification Results after 20 Rounds of Sample Division

**Figure 8:**
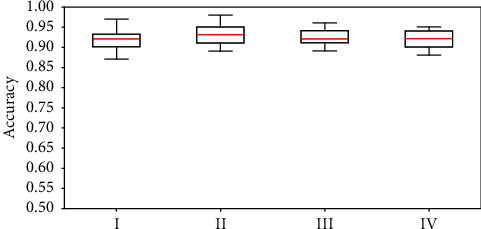
Blot plots for making a comparison among ensemble classification results using the selected 20 variables (I), using all the 108-dimensional features (II), L-SHADE-SVM-SVD using all the 108-dimensional features (III), and L-SHADE-SVM-SVD only using the first 64 components (IV). The red lines refer to the median values.

In order to diminish the effect of random sample selection on evaluating the predictive capability, we repeat the whole procedure shown in Figure 1 for 20 times. The accuracy, precision, Recall, and *F*1 measure are calculated as expressed in equations (6) and (7), in order to make a quantitative comparison with the experimental results of L-SHADE-SVM-SVD [17] on each 10% of the independent testing set. The experimental results are illustrated in Table 1 and Figure 8, respectively. It can be seen that the selected 20 variables using our variable selection strategy obtain better classification results on automatic classification of concrete surface voids among images. As seen in Table 1, our method on all the 108 variables keeps the highest accuracy, precision, and *F*1-measure mean values. Besides, our method on the selected 20 variables keeps the highest recall mean value and comparable other values. Yet, L-SHADE-SVM-SVD using all the 108 variables keeps most of the lowest standard deviations. This phenomenon can also be seen in Figure 8.

**Figure 9:**
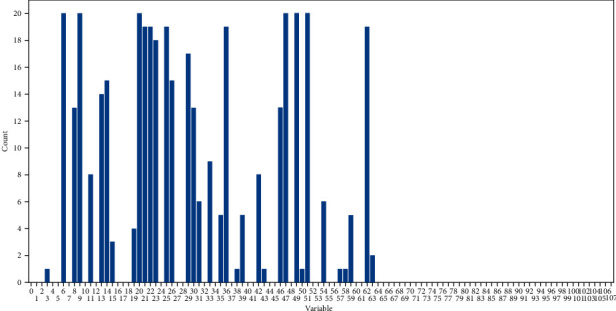
A histogram which represents the count of each variable selected during the 20 rounds of sample division.

Moreover, we record the selected 20 variables by adding one point to each component. After 20 times of sample division, a histogram representing the counts of every variable is obtained and shown in Figure 9. The variables with higher counts indicate better interpretation to classification results. It can be seen that the important variables focus on the first 64 components of the 108 ones.

## 4. Discussion

Experimental results have indicated the effectiveness of variable selection from image texture feature. In this section, we will further discuss three important facts derived from the experimental results.

Firstly, we wonder whether only some components but not the whole feature may work. Actually, it has been confirmed by experimental results shown in Figure 5 and Table 1. In Figure 5, it can be seen that the selected 20 variables keep a high accuracy comparable to that of the 108-dimensional feature. This phenomenon is further emphasized after the comparison between the first two columns in Table 1.

Secondly, it needs to be discussed whether various classifiers and their parameters are to be tried in order to get better classification accuracies. Unlike L-SHADE-SVM-SVD, our approach only utilized the default parameters of seven classifiers without any optimization. Anyway, better classification results are shown using our method (see Table 1 and Figure 8). Besides, it can be indicated from the pan chart shown in Figure 3 that LR and kNN may be the more proper classifiers getting at least the same or even better classification results as L-SHADE-SVM-SVD does.

Last but most importantly, feature interpretability needs to be discussed. In Figure 9, the counts of each variable derived from an accumulation of first 20 selections in each round are labeled using a histogram. Interestingly, the labeled variables with positive counts only appear in the first 64 components of the 108-dimensional texture feature, which indicates the texture feature expressed in equation (3) to be redundant. In order to confirm this phenomenon, L-SHADE-SVM-SVD is performed using the 64 components derived from Gabor Filter response only. The corresponding experimental results are shown in Figure 8 (group IV), which indicate that there is no need to use gray-level run lengths. In addition, variables with high counts in Figure 9 are considered to be interpretable. Anyway, each variable represents which one of the statistics shown in equation (4) with what certain orientation angle and radial frequency is still unclear due to lack of information in the provided image samples of the concrete surface.

## 5. Conclusions

In this paper, we propose a variable selection method for automatic classification of concrete surface voids. The image texture feature deriving from the Gabor filter and gray-level run lengths is employed. A variable selection strategy with seven steps is presented and utilized on the 108 components of the feature. Using the 1000 provided image samples, important variables are automatically selected to build ensemble classifiers for accurate classification of concrete surface voids. Feature interpretability is also discussed. The Gabor Filter is viewed as a stable source to provide texture feature. In the next stage, the major task is to discover further interpretability of the statistics with various orientation angles and radial frequencies derived from the Gabor Filter response.

## Figures and Tables

**Table 1:** Prediction result comparison on the independent testing sets.

Method	Proposed method on 20 selected variables		Proposed method on 108 variables		L-SHADE-SVM-SVD on 108 variables	
	Mean	Std.	Mean	Std.	Mean	Std.
Acc	0.919	0.026	**0.929**	0.025	0.926	**0.018**
Precision	0.904	0.044	**0.922**	0.042	**0.922**	**0.025**
Recall	**0.938**	0.034	0.937	**0.029**	0.932	0.037
*F*1 measure	0.920	0.027	**0.929**	0.027	0.926	**0.018**

## Data Availability

Data provided by Hoang and Nguyen [17] for comparison can be downloaded from the repository of github (https://github.com/NhatDucHoang/L-SHADE-SVM-SVD).

## References

[1] Haber R. E., Alique J. R. (2007). Fuzzy logic-based torque control system for milling process optimization. *IEEE Transactions on Systems, Man and Cybernetics, Part C (Applications and Reviews)*.

[2] Ramírez M., Haber R., Pena V., Rodriguez I. (2004). Fuzzy control of a multiple hearth furnace. *Computers in Industry*.

[3] Guerra R. H., Quiza R., Villalonga A., Arenas J., Castano F. (2019). Digital twin-based optimization for ultraprecision motion systems with backlash and friction. *IEEE Access*.

[4] Castano F., Beruvides G., Haber R. E., Artunedo A. (2017). Obstacle recognition based on machine learning for on-chip lidar sensors in a cyber-physical system. *Sensors*.

[5] Liu B., Yang T., Xie Y. (2017). Factors influencing bugholes on concrete surface analyzed by image processing technology. *Construction and Building Materials*.

[6] Laofor C., Peansupap V. (2012). Defect detection and quantification system to support subjective visual quality inspection via a digital image processing: a tiling work case study. *Automation in Construction*.

[7] Ozkul T., Kucuk I. (2011). Design and optimization of an instrument for measuring bughole rating of concrete surfaces. *Journal of the Franklin Institute*.

[8] Lemaire G., Escadeillas G., Ringot E. (2005). Evaluating concrete surfaces using an image analysis process. *Construction and Building Materials*.

[9] Kalayci A. S., Yalim B., Mirmiran A. (2009). Effect of untreated surface disbonds on performance of FRP-retrofitted concrete beams. *Journal of Composites for Construction*.

[10] Ichimiya K., Idemitsu T., Yamasaki T. (2002). The influence of air content and fluidity of mortar on the characteristics of surface voids in self-compacting concrete. *Doboku Gakkai Ronbunshu*.

[11] Paul S. (1970). Voids in concrete surfaces. *Asian College of Journalism*.

[12] Reading T. J. (1972). The bughole problem. *Asian College of Journalism*.

[13] Thompson M. S. (1969). Blowholes in concrete surface. *Concrete*.

[14] Liu B., Yang T. (2017). Image analysis for detection of bugholes on concrete surface. *Construction and Building Materials*.

[15] Zhu Z., Brilakis I. (2010). Machine vision-based concrete surface quality assessment. *Journal of Construction Engineering and Management*.

[16] da Silva W. R. L., Štemberk P. (2013). Expert system applied for classifying self-compacting concrete surface finish. *Advances in Engineering Software*.

[17] Hoang N., Nguyen Q. (2020). A novel approach for automatic detection of concrete surface voids using image texture analysis and history-based adaptive differential evolution optimized support vector machine. *Advances in Civil Engineering*.

[18] Cha Y.-J., Choi W., Büyüköztürk O. (2017). Deep learning-based crack damage detection using convolutional neural networks. *Computer-aided Civil and Infrastructure Engineering*.

[19] Yao G., Wei F., Yang Y., Sun Y. (2019). Deep-learning-based bughole detection for concrete surface image. *Advances in Civil Engineering*.

[20] Wei F., Yao G., Yang Y., Sun Y. (2019). Instance-level recognition and quantification for concrete surface bughole based on deep learning. *Automation in Construction*.

[21] Kim N. C., So H. J. (2018). Directional statistical Gabor features for texture classification. *Pattern Recognition Letters*.

[22] Jo J., Jadidi Z. (2019). A high precision crack classification system using multi-layered image processing and deep belief learning. *Structure and Infrastructure Engineering*.

[23] Chu A., Sehgal C. M., Greenleaf J. F. (1990). Use of gray value distribution of run lengths for texture analysis. *Pattern Recognition Letters*.

[24] Zhang J., Lv L. X., Lu D. L., Kong D. N., Al-Alashaari M. A. A., Zhao X.D. (2020). Variable selection from a feature representing protein sequences: a case of classification on bacterial type IV secreted effectors. *BMC Bioinformatics*.

[25] Liu T., Li H., Zhao X. (2019). Clustering by search in descending order and automatic find of density peaks. *IEEE Access*.

[26] Zhao X. D., Jiao Q., Li H. Y., Wu Y. M., Wang H. X., Wang G. H. (2020). ECFS-DEA: an ensemble classifier-based feature selection for differential expression analysis on expression profiles. *BMC Bioinformatics*.

